# Digital breast tomosynthesis compared to diagnostic mammographic projections (including magnification) among women recalled at screening mammography: a systematic review for the European Commission Initiative on Breast Cancer (ECIBC)

**DOI:** 10.1002/cam4.3803

**Published:** 2021-03-05

**Authors:** Carlos Canelo‐Aybar, Lourdes Carrera, Jessica Beltrán, Margarita Posso, David Rigau, Annette Lebeau, Axel Gräwingholt, Xavier Castells, Miranda Langendam, Elsa Pérez, Paolo Giorgi Rossi, Ruben Van Engen, Elena Parmelli, Zuleika Saz‐Parkinson, Pablo Alonso‐Coello

**Affiliations:** ^1^ CIBER de Epidemiología y Salud Pública (CIBERESP) Madrid Spain; ^2^ Department of Clinical Epidemiology and Public Health Biomedical Research Institute Sant Pau (IIB Sant Pau) Barcelona Spain; ^3^ Universidad Nacional Mayor de San Marcos Lima Perú; ^4^ Department of Epidemiology and Evaluation Hospital del Mar Medical Research Institute (IMIM) Barcelona Spain; ^5^ Institute of Pathology University Medical Center Hamburg‐Eppendorf Hamburg Germany; ^6^ Radiologie am Theater Paderborn Germany; ^7^ Department of Clinical Epidemiology, Biostatistics and Bioinformatics Amsterdam UMC University of Amsterdam Amsterdam Public Health Institute Amsterdam The Netherlands; ^8^ University Hospital Dr. Josep Trueta Girona Spain; ^9^ Epidemiology Unit Azienda USL – IRCCS di Reggio Emilia Reggio Emilia Italy; ^10^ LRCB, Dutch Expert Centre for Screening Nijmegen The Netherlands; ^11^ European Commission Joint Research Centre (JRC) Ispra Italy

**Keywords:** breast neoplasms, digital breast tomosyntheses, mass screening, practice guidelines, systematic review

## Abstract

**Background:**

Diagnostic mammography projections (DxMM) have been traditionally used in the assessment of women recalled after a suspicious screening mammogram. Digital breast tomosynthesis (DBT) reduces the tissue overlap effect, thus improving image assessment. Some studies have suggested DBT might replace DxMM with at least equivalent performance.

**Objective:**

To evaluate the replacement of DxMM with DBT in women recalled at screening.

**Methods:**

We searched PubMed, EMBASE, and the Cochrane Library databases to identify diagnostic paired cohort studies or RCTs comparing DBT vs DxMM, published in English that: reported accuracy outcomes, recruited women recalled for assessment at mammography screening, and included a reference standard. Subgroup analysis was performed over lesion characteristics. We provided pooled accuracy estimates and differences between tests using a quadrivariate model. We assessed the certainty of the evidence using the GRADE approach.

**Results:**

We included ten studies that reported specificity and sensitivity. One study included 7060 women while the remaining included between 52 and 738 women. DBT compared with DxMM showed a pooled difference for the sensitivity of 2% (95% CI 1%–3%) and a pooled difference for the specificity of 6% (95%CI 2%–11%). Restricting the analysis to the six studies that included women with microcalcification lesions gave similar results. In the context of a prevalence of 21% of breast cancer (BC) in recalled women, DBT probably detects 4 (95% CI 2–6) more BC cases and has 47 (95%CI 16–87) fewer false‐positive results per 1000 assessments. The certainty of the evidence was moderate due to risk of bias.

**Conclusion:**

The evidence in the assessment of screen‐recalled findings with DBT is sparse and of moderate certainty. DBT probably has higher sensitivity and specificity than DxMM. Women, health care providers and policymakers might value as relevant the reduction of false‐positive results and related fewer invasive diagnostic procedures with DBT, without missing BC cases.

## Graphical abstract

This systematic review found that in women of average risk of breast cancer with suspicious lesions at mammography screening, DBT probably has higher specificity difference (6%) than diagnostic views of 2D mammography in women recalled for assessment (moderate certainty evidence). The sensitivity is also probably higher with DBT; however, the difference was not so large (2%). The clinical impact of these results in a screening program is a probable reduction of 47 false positive results, including related invasive procedures, and most likely a small increase of 4 additional true BC lesions per 1000 women recalled for assessment. Our results were consistent in women with calcified lesions, but with a larger imprecision in the sensitivity differences. These results were taken into account by the GDG, together with other considerations (ie, feasibility) to issue a conditional recommendation in favor of DBT which is available on the ECIBC website (https://healthcare‐quality.jrc.ec.europa.eu/european‐breast‐cancer‐guidelines/diagnosis/DBT).

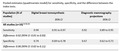

## 1 INTRODUCTION

Breast cancer (BC) is the second most prevalent cancer in the world and the most frequent among women. In the European Union, 404,920 women were diagnosed with breast cancer in 2018.^1^ BC mortality has decreased over the last decades, partially due to the implementation of mammography screening programs,^2^, ^3^ which are recognized as an effective method to detect early‐stage breast cancers.^4^ Consequently, the European Breast Guidelines on Screening and Diagnosis recommends mammography screening for asymptomatic women aged 50–69, with an average risk of BC, and suggests mammography screening for women aged 45–49 and 70–74.^5^, ^6^

When an abnormality is found at mammography screening, women are recalled for assessment, which might mean they need to undergo additional imaging testing. Mammography for assessment of suspicious abnormalities also referred to as “diagnostic mammography projections,” usually consists of additional mammographic views (ie, spot compression, Cleopatra view, cleavage view, among others) or magnification if two‐view mammography from the previous screening examination is available. During this imaging assessment, the suspicious finding from mammography screening can be either confirmed, in a minority of women who are then referred to an invasive assessment to obtain a sample of tissue or cells,^7^ or not confirmed, and the woman is usually returned to a new screening round. Recall for further invasive assessment (ie, biopsy) leads to additional costs and anxiety, not only during the diagnostic work‐up but also through subsequent screening mammogram rounds (despite a negative result).^8^

Digital breast tomosynthesis (DBT) is a mammographic technique that acquires low‐dose projection images of the breast at different angles utilizing a moving X‐ray source. A stack of thin slices is reconstructed, overcoming the influence of overlapping breast tissue.^9^ DBT improves the visualization of BC and thus may enhance the interpretation of mammography. Several studies suggest that DBT may be a promising technique as a screening modality.^10^ In the “further assessment” setting, however, the evidence is scarce. Li et al. reviewed the evidence of DBT compared to conventional imaging in the assessment of screen‐recalled findings, reporting limited evidence for a higher specificity of DBT.^11^

In 2015, the European Commission Initiative on Breast Cancer (ECIBC) was launched to develop the European Guidelines on Breast Cancer Screening and Diagnosis.^5^ This systematic review informed the recommendations on the use of digital breast tomosynthesis (DBT) compared to diagnostic mammography projections (DxMM), for the assessment of suspicious abnormalities in women recalled for further assessment at mammography screening in average risk asymptomatic women. During the guideline´s development process,^5^ the Guidelines Development Group (GDG) made detailed considerations on the evidence to decide about the balance between desirable and undesirable effects of the interventions to issue a recommendation. The GDG also considered other criteria such as values and preferences, equity, acceptability, and feasibility while upholding independence of commercial, private, and national interests. We encourage readers to refer to these detailed considerations in the published recommendations on the ECIBC website (https://healthcare‐quality.jrc.ec.europa.eu/european‐breast‐cancer‐guidelines/diagnosis/DBT)

## 2 METHODS

### 2.1 Guidelines development group (GDG)

The European Commission selected, via an open call, a panel with a broad representation of different expertise, knowledge, and background, that includes patients, healthcare professionals, epidemiologists, guideline methodologists, and others (the complete list of experts is available from the ECIBC website) who voluntarily participate in the ECIBC.

### 2.2 Structured question and outcome prioritization

The clinical question “Should *digital breast tomosynthesis (DBT) vs. diagnostic mammography projections (including magnification) be used in the assessment of recalled women of average risk of breast cancer due to suspicious lesions at mammography screening?*” was prioritized by the GDG, and here, we describe the test accuracy assessment. The question was structured following a diagnostic framework format of Population, Index test, Comparator (standard index test), Target condition, and Reference standard (Box 1).

BOX 1 Clinical question definition for assessment of accuracy between two tests*

**Table:** 

**Population**	**Index test**	**Comparison test**	**Reference standard**	**Target condition**
Women of average risk for breast cancer with abnormal findings at mammography screening who are recalled for further imaging assessment	DBT images (one or two views), including either synthesized mammography or the previous screening mammography that triggered the assessment	Diagnostic mammography projections or magnification	Histopathological exam and/or imaging follow‐up for at least one year	Breast cancer lesions (DCIS or invasive)

DBT, digital breast tomosynthesis; DCIS, ductal carcinoma in situ.

* Both tests could have been interpreted with or without the additional help of breast ultrasound or clinical examination.

This diagnostic question was framed as a replacement scenario among women of average risk of BC who have any abnormality at mammography screening that leads to a recall for assessment. These women are usually examined with further diagnostic mammography views (including magnifications), but these may be replaced with DBT images. Both index tests (DBT or DxMM) can be performed alongside with other exams if required (ie, clinical examination, ultrasound).

### 2.3 Eligibility criteria

Studies were included if: (1) they compared the accuracy of DBT images (one or two views), including either synthesized mammography or the previous screening mammography that triggered the assessment, with diagnostic mammography projections or magnification; (2) participants were women of average risk for BC with abnormal findings at mammography screening; (3) there was a minimum sample size of 30 participants; (4) the study provided enough data to construct a two by two contingency table; (5) all participants either received both DBT and diagnostic mammography projections (or magnification) or were randomly allocated to the index comparison tests; (6) a histopathological examination and/or imaging follow‐up for at least one year was used as a reference standard.

Included cohort studies could either prospectively recruit and assess women recalled at screening or perform a blind re‐interpretation of the two index tests from retrospective case series that had both tests recorded during the assessment. Both index tests could have been interpreted with or without the additional help of breast ultrasound or clinical examination. The following exclusion criteria were applied: (1) studies reporting DBT performed as a screening test in the general population; (2) diagnostic studies of case‐control design or non‐paired cohorts studies; (3) abstracts or conference communications not published as complete articles, and; (4) studies published in a language other than English.

All citations retrieved were imported into a bibliographic reference software (EndNote X5; Thomson Reuters) to discard duplicates, and record screening decisions. Initially, at the title and abstract level, two previously calibrated reviewers (CCA and LC) assessed eligibility. In a second step, two reviewers independently reviewed the full text of all selected references. Discrepancies were solved either by discussion or with the help of a third reviewer (DR).

### 2.4 Data sources and searches

We searched MEDLINE (via PubMed, May 2018), EMBASE (via Ovid, May 2018), and CENTRAL (via The Cochrane Library, May 2018) databases using predefined algorithms for individual studies. We adapted the search terms to each database and used validated filters to retrieve appropriate designs. Additionally, we updated our initial search in MEDLINE (via PubMed) and EMBASE (via Ovid) in February 2020 (Table S1 *: Search strategy*). We also reviewed lists of references of the included studies, and members of the GDG were consulted about potential missing studies.

### 2.5 Data extraction and risk of bias assessment

Two reviewers (LC, CCA) independently extracted data and assessed risk of bias from the included studies. We collected the following information from each study: accuracy results (TP, TN, FP, and FN), the total number of participants, country, year of publication, first screening modalities, type of suspicious lesions that triggered the recall for assessment (ie, masses, architectural distortions, asymmetry, calcifications), participants´ mean age, study design, characteristics of the index tests at assessment (ie, the number of views, type of additional views, use of additional tests), classification system, and cutoff used to interpret the index test´s results (ie, BI‐RADS), and type of reference standard used. Discrepancies were solved either by discussion or with the help of a third reviewer (DR).

We assessed the risk of bias of the included studies using the Quality Assessment of Diagnostic Accuracy Studies (QUADAS‐2) tool^12^ which includes the following four domains: patient selection, index test, reference standard, flow, and timing. To adapt the QUADAS‐2 tool to the comparison of two index tests, we also assessed whether: (1) a random allocation of participants to each index test or paired comparison was implemented; (2) radiologists reading any of the index tests were blinded to the results of the alternative test or the reference standard; (3) the timing elapsed between the examination with one index test and the other was less than 1 month; 4) if there were no unbalanced number of additional examinations performed together with the index tests; (5) the reference standard was the same for both index tests, even if studies might have used an incomplete verification (ie, biopsies for positive results but clinical follow‐up for negative ones).

### 2.6 Data analysis

We classified the index test results as positive or negative according to the reporting system used in each study. When we were able to reconstruct the 2 by 2 contingency table based on disaggregated data, we considered undetermined results as positive, as they imply recalling the women for further assessment (ie, BI‐RADS 0). If results were provided for multiple readers within a study, readers´ data were averaged before analysis.

To perform a meta‐analysis of diagnostic tests, the current recommended method is the bivariate random‐effects model. This model takes into account the correlation between sensitivity and specificity at the study level, as well as underlying variability across studies due to difference in the (implicit) thresholds used to interpret index test results or to the studies’ designs.^13^ In this review, we implemented a quadrivariate generalized linear mixed random model (GLMM). This is an extension of the bivariate model described before, to jointly account for the sensitivities and specificities parameters of two diagnostic tests (and the correlation between them), compared to a common reference standard.^14^

We present the hierarchical summary receiver operating characteristic curves (HSROC) using the model parameters from the bivariate model for each index test with the “madas” package in RStudio 3.5.1. We used the graphical functions of the *metafor* package in RStudio to display the forest plots for sensitivity and specificity, as well as their differences. We did not statistically evaluate publication bias as there is currently no accepted method for comparative test analysis.

We assessed heterogeneity by visual inspection of forest plots, as statistical approaches (ie, I‐squared percentage) may overestimate the variability across studies and do not consider the clinical relevance of the results. Predefined subgroup analysis included: risk of bias (low versus high), type of further mammographic assessment (magnification vs additional views), use of additional ultrasound in either index test, the extension of microcalcification, and type of system used to classify the index test results (BI‐RADS vs others).

All statistical analyses were performed in SAS University Edition, using the PROC GLIMMIX with a penalized quasi‐likelihood (PQL) method, a logit link, and the Newton‐Raphson Ridge Optimization technique. Due to the non‐convergence of the quadrivariate model with sparse data, we only performed a sensitivity analysis without the studies that explicitly excluded women with calcified lesions.

### 2.7 Certainty of the evidence

We rated the overall certainty of the evidence as high, moderate, low or very low according to the GRADE Working group´s guidance for the assessment of accuracy studies which includes the following domains: risk of bias, imprecision, inconsistency, indirectness, and publication bias.^15^ We considered a comparative accuracy approach to rate the certainty of evidence (ie, the heterogeneity was not assessed for a single index test but relative to the comparator index test).

## 3 RESULTS

### 3.1 Search result

**FIGURE 1:**
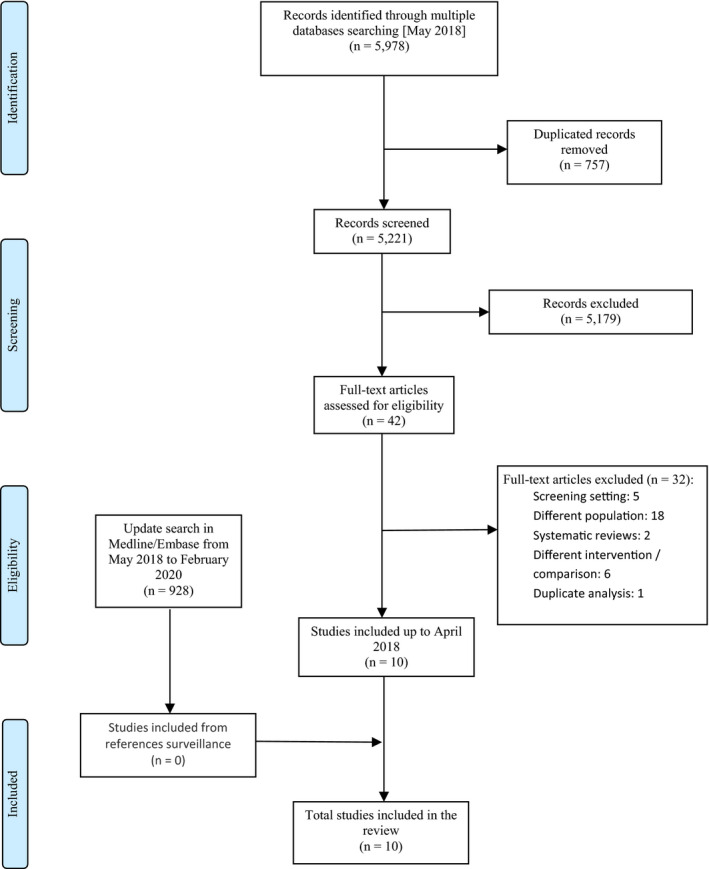
Flow chart for the evidence of effects.

A total of 5,978 unique citations were initially retrieved. We excluded 757 duplicate records together with 5,179 citations based on title or abstract review and selected 42 for a detailed appraisal of the full text (Figure 1). We excluded 32 studies that either: included a different population (symptomatic women, screening population, or invasive BC under staging assessment), reported a different comparison to diagnostic mammography (eg, ultrasound), or used DBT in the screening setting (see supplementary file Table S3). In total, we included 10 primary accuracy studies,^9^, ^24^ of which nine were designed as prospective cohorts^9^, ^17^, ^18^, ^19^, ^20^, ^21^, ^22^, ^23^, ^24^, and one was a blinded re‐assessment of retrospective case series imaging from several breast screening centers.^16^ Finally, after the update search in February 2020, we did not identify any additional studies that fulfilled our eligibility criteria (see supplementary file Table S3).

### 3.2 Study characteristics

The included studies were conducted in the United States of America, Italy, Germany, Switzerland, and the United Kingdom. Five studies did not describe the age of the included women,^9^, ^20^, ^22^, ^23^, ^24^ The mean age of women was between 51 and 58 in three studies,^16^, ^19^, ^21^ and the age range was between 50 and 69 in another one.^18^ One study stated that only women older than 40 were included.^20^ Women were recalled for assessment after an abnormal finding during screening with digital mammography in five studies and film mammography in one study, while in four studies the type of screening mammography was not described. One study had a much higher sample size (n = 7060),^16^ while the number of women recalled for assessment in the remaining studies was between 52^19^ and 738^9^ (Table 1).

**TABLE 1:** Characteristics of the included studies in the systematic review

**Author (year), country**	**Number of patients**	**Age** **Mean (range)**	**Type of lesions**	**Digital breast tomosynthesis**	**Diagnostic mammography projections**	**Index test threshold**	**Number of radiologists and experience**	**Reference test**
Brandt (2013),^17^ US	146	>40	‐Possible masses 20% ‐Areas of distortion 18% ‐Asymmetries 63% ‐Calcifications: excluded	Two‐view DBT Ultrasound in selected cases	May include digital spot compression (with or without magnification), rolled views, lateral views, exaggerated views Ultrasound in selected cases	Positive: BI‐RADS 4, 5 Negative: BI‐RADS 1 ‐ 3	DBT: three independent readers* with 8 hours‐training 2DM: ND	Positive lesions: biopsy‐proven cancers or high‐risk abnormality Negative: biopsy‐proven benign, or no BC at follow‐up
Cornford (2016),^20^ UK	324	ND	‐ Masses (50%) ‐ Distortions (13.5%) ‐ Asymmetric densities (36.5%) ‐ Microcalcification (3.8%)	Two views DBT	May include extended CC views, lateral projections or magnification views	Positive: UK‐RCS M3‐M5 Negative: UK‐RCS M1‐M2	8 specialist Brest radiologists. (7‐24 years of experience) DBT and 2DM: 1‐day training in interpretation of DBT images (blinded)	Biopsy lesion histopathology.
Gilbert (2015),^16^ UK	7,060	56 (29‐ 85)	‐ Mass (69%) ‐ Microcalcifications (13%) ‐ Distortion or ASD (18%)	Two views DBT (plus synthesized mammography)	May include magnification	Positive: “reader decided to recall” Negative: “reader decided not to recall”	Average of 10 years of experience (range 3‐25)	Biopsy lesion histopathology and imaging re‐assessment before discharge
Heywangkbrunner (2017)^a^,^18^ Germany	284 patients with 311 lesions	50–69 years	Screen‐detected abnormalities that needed further mammographic assessment.	Single view with a wide‐angle DBT view CC projection for lesions not visible in one view	May include 1 or 2 mammographic additional views Ultrasound in selected cases	Positive: BI‐RADS 0, 3‐5 Negative: BI‐RADS 1,2	Consensus reading Reader 1 (the principal investigator) Reader 2 experience with DBT on > 200 proven cases.	BIRAD 4 OR 5 histopathological proof and 2‐year follow‐up
Heywangkbrunner (2018),^24^ Germany	241	ND	Cases with microcalcifications were excluded	Single view with a wide‐angle DBT view CC projection for lesions not visible in one view Ultrasound in selected cases	May include additional views Ultrasound in selected cases	Positive: BI‐RADS 0, 3‐5 Negative: BI‐RADS 1, 2	Blinded reading	Needle biopsy Histology and follow‐up > 2 years
Michell (2012),^9^ UK	738	ND	Mammographic abnormality: circumscribed, spiculate, microcalcifications (21%), parenchymal distortion, asymmetry.	Two views	May include magnification, black/with inversion (for both exams)	Positive: UK‐RCS M3‐M5 Negative: UK‐RCS M1‐M2	One specialist breast radiologist	Positive: final surgical histology. Negative: benign findings in needle biopsy
Poplack (2007) ^a^,^23^ US	100	ND	Abnormal digital screening mammogram interpreted prospectively	Up to 3 projections resembling the mammography exam	May include focal compression (film mammography) Ultrasound	Results reported by BI‐RADS categories	Seven mammography technologists trained to perform tomosynthesis examinations before the study.	Core needle biopsy was performed with stereotactic guidance
Tagliafico (2012),^19^ Italy	52	51 ± 12	Women with BI‐RADS 0 lesions (calcifications were excluded)	Single view	Spot compression:	Positive: BI‐RADS 3‐5 Negative: BI‐RADS 1, 2	Blinded two experienced breast radiologists with 20 and 7 years of experience.	Ultrasound + fine needle aspiration and core biopsy + follow > 12 months
Waldherr (2013),^22^ Switzerland	66	ND	Abnormalities at the screening included: masses, microcalcifications	Single view	ND	Positive: BI‐RADS 4‐5 Negative: BI‐RADS 1‐3, 0	Blinded reading	Histopathology + follow ‐up for 12–16 months
Whelehan (2017),^21^ Germany	238	57.6 (50‐69)	Microcalcifications were excluded	Single view	Supplementary views	Positive: biopsy requested Negative: biopsy not requested	2DM: 10.9 mean years of experience. DBT: 1.5 mean years of experience	Histopathology + follow ‐up for two years

AV, additional views; ND, not described; DBT, digital breast tomosynthesis; BI‐RADS, breast imaging reporting and data system; UK‐RCS, UK royal College of Radiologist score.

^a^ Data were categorized as positive (BI‐RADS 0, 3‐5) and as negative BI‐RADS (1 and 2).

All studies enrolled women recalled from screening mammography with abnormalities such as masses, areas of distortion, asymmetries, or other non‐calcified lesions.^9^, ^24^ Five studies also included women with calcified lesions,^9^, ^16^, ^20^, ^22^ but two of them did not report the exact proportion of patients with calcifications,^18^, ^22^ and another one had a low proportion (3.8%) because women with calcifications as the predominant lesion were not invited to participate in the study.^20^

Five studies used a two‐view DBT,^9^, ^16^, ^17^, ^20^, ^23^ while the other five used a single view DBT during the assessment.^18^, ^19^, ^21^, ^22^, ^24^ Diagnostic mammography projections included additional or supplementary views such as digital spot compression views, rolled view, lateral views with or without magnification. One study did not describe what type of supplementary views was used.^22^ Ultrasound was also used in five studies either to assess selected cases at the clinician discretion or in the assessment of all cases included.^17^, ^18^, ^20^, ^23^, ^24^

In six studies, the screening mammography images used for recall were available during the DBT assessment.^9^, ^17^, ^18^, ^20^, ^21^, ^24^ In three studies the readers either based their assessment on DBT alone or did not clearly describe if they had access to the previous mammography images.^19^, ^22^, ^23^ One study interpreted the DBT images together with synthesized mammography,^16^ which was compared to a DxMM assessment that included magnification views if women had microcalcifications.

Regarding the classification system used to interpret the DBT and mammography results, six studies used the breast imaging reporting and data system (BI‐RADS). Three of these defined positive result as BI‐RADS of 3 to 5 (two of them including BI‐RADS 0).^18^, ^19^, ^24^ Two studies considered positive results as BI‐RADS 4 to 5,^17^, ^22^ and one reported the results according to each BI‐RADS category.^23^ The UK Royal College of Radiologist score (UK‐RCS) was used in two studies and categorized results as positive if classified between UK‐RCS M3 to M5.^9^, ^20^ In the remaining two studies, a result was considered positive if recall or biopsy was requested by the imaging assessor.^16^, ^21^

The reference standard also differed across studies but not between the index tests. Six studies included a clinical or imaging follow‐up for negative results at assessment^17^, ^18^, ^19^, ^21^, ^22^, ^24^ which lasted between 12 months^19^ to more than two years^18^. In the remaining four studies, results were confirmed based only on histopathological results^9^, ^20^, ^23^ or together with re‐assessment of the breast images before discharge.^16^

### 3.3 Sensitivity

**FIGURE 2:**
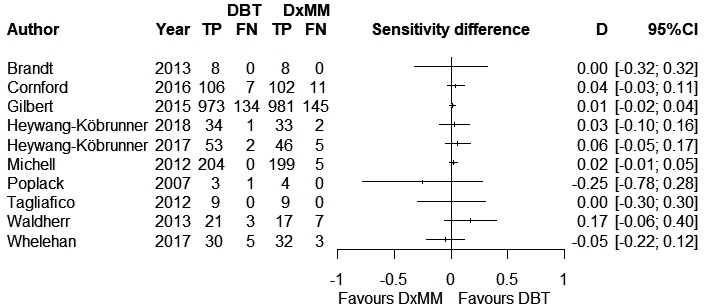
Forest plots for the difference in sensitivity comparing digital breast tomosynthesis vs. diagnostic mammography in recalled women.

A total of 1,592 cases of BC lesions were included in the analysis. The sensitivity difference between DBT and DxMM ranged from −25%^23^ to 17% across studies, while six studies showed a probably higher sensitivity for DBT. The visual assessment of the forest plots for each test independently did not show a relevant heterogeneity for either DBT or DxMM (Figure S1). A relatively homogenous pattern was also observed when the sensitivity difference was plotted (Figure 2).

The pooled sensitivity was 94% (95% CI 91–97%) for DBT, and 92% (95% CI 89–95%) for DxMM. The pooled difference was 2% (95% CI 1–3%) in favor of DBT (*p *< 0.001) (Table 2). For a BC prevalence of 21% in women recalled for assessment, DBT probably identifies 4 more (95% from 2 to 6 more) true BC cases per 1000 assessments (Table 3).

**TABLE 2:** Pooled estimates (quadrivariate model) for sensitivity, specificity, and the difference between the index tests.

Population (N of studies)	Digital breast tomosynthesis		Diagnostic mammography projections	
		95% CI		95% CI
**All studies (*n* = 10)** ^9^, ^24^				
Sensitivity	0.94	0.91–0.97	0.92	0.89–0.95
Difference: 0.02 (95% CI 0.01–0.03)				
Specificity	0.74	0.69–0.78	0.67	0.62–0.73
Difference: 0.06 (95% CI 0.02–0.11)				
**Studies including calcified lesions** ^a^ **(*n* = 6)** ^9^, ^16^, ^18^, ^20^, ^22^, ^23^				
Sensitivity	0.94	0.90–0.97	0.89	0.84–0.93
Difference: 0.05 (95% CI 0.01–0.09)				
Specificity	0.71	0.66–0.75	0.60	0.54–0.65
Difference: 0.12 (95% CI 0.06–0.17)				
**Calcified lesions only** ^b^ **(*n* = 1)** ^16^				
Sensitivity	0.85	0.81–0.89	0.88	0.84–0.92
Difference: ‒0.03 (95% CI −0.09 to −0.03)				
Specificity	0.44	0.40–0.48	0.31	0.28–0.34
Difference: 0.13 (95% CI 0.08–0.18)				

*n* = number of studies included in the analysis.

^a^ Studies that included women with or without calcified lesions.

^b^ Subgroup analysis of women with pure calcified lesions. The estimate was calculated using a standard difference of proportions formula for a single comparison.

**TABLE 3:** Summary of findings (GRADE) for digital breast tomosynthesis versus diagnostic mammography. *Pooled sensitivity digital breast tomosynthesis: 0.94 (95% CI: 0.91–0.97) | Pooled specificity digital breast tomosynthesis: 0.74 (95% CI: 0.69–0.78). Pooled sensitivity assessment mammography: 0.92 (95% CI: 0.89–0.95) | Pooled specificity assessment mammography: 0.67 (95% CI: 0.62–0.73). Pooled sensitivity difference: 0.02 (95% CI: 0.01–0.03) | Pooled specificity difference: 0.06 (95% CI: 0.02–0.11)^#^

Test result	Number of results per 1000 patients tested (95% CI)		Number of participants (studies)	Certainty of the Evidence (GRADE)
	Prevalence 21% Typically seen in European screening programs			
	Digital breast tomosynthesis	Diagnostic mammography		
True positives	197 (191–204)	193 (187–199)	1584 (10)^9^, ^24^ ^, a^	⨁⨁⨁◯ MODERATE ^b^, ^c^, ^d^, ^e^, ^f^, ^h^
	4 more (from 2 more to 6 more) TP in digital breast tomosynthesis			
False negatives	13 (6–19)	17 (11–23)		
	4 fewer (from 2 fewer to 6 fewer) FN in digital breast tomosynthesis			
True negatives	585 (545–616)	529 (490–577)	6096 (10) ^9^, ^24^ ^, a^	⨁⨁⨁◯ MODERATE ^b^, ^e^, ^f^, ^g^, ^h^
	47 more (from 16 more to 87 more) TN in digital breast tomosynthesis^#^			
False positives	205 (174–245)	261 (213–300)		
	47 fewer (from 16 fewer to 87 fewer) FP in digital breast tomosynthesis^#^			

Abbreviations: CI, confidence interval; TP, true positives; FN, false negative; TN, true negative; FP, false positive.

^a^ The absolute differences are the additional cases identified or missed with digital breast tomosynthesis compared to diagnostic mammographic views, among those women, recalled at the screening mammography assessment.

^b^ In some of the included studies, there was a non‐blinded reading of the index tests. There was variability in how the evaluations were performed; in some cases, they included additional tests such as ultrasound. Those additional exams could be requested at clinical discretion and therefore could be a potential source of differential misclassification in the test accuracy estimates.

^c^ There was no important unexplained heterogeneity in the sensitivity difference between DBT and DxMM

^d^ One study (Poplack 2012) showed largely inconsistent results. However, a sensitivity analysis excluding this study did not show relevant differences in the pooled estimate.

^e^ One study (Gilbert 2015), performed a retrospective analysis with DBT plus synthesized two‐view mammography as the intervention index test. In the remaining studies, the intervention index test was DBT plus screening mammography.

^f^ Publication bias was not suspected by the expert panel.

^g^ There was no important unexplained heterogeneity in the specificity difference between DBT and DxMM.

^h^ Observed variability might be explained using different thresholds to define positivity, use of diverse additional imaging tests, different reference standards and the imprecision of the estimates. The panel decided not to downgrade the certainty of evidence due to this variability.

* The SoF table have been adapted *ad hoc* for a two index test comparison.

# Due to rounding the estimated specificity difference might differ from the manual subtraction between each test´s estimates.

### 3.4 Specificity

**FIGURE 3:**
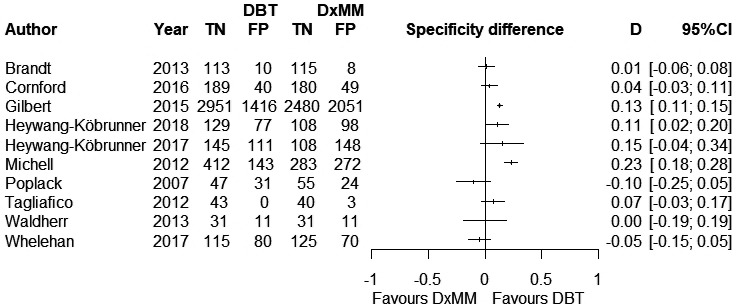
Forest plots for the difference in specificity comparing digital breast tomosynthesis vs. diagnostic mammography in recalled women.

A total of 6096 negative cases were included in the analysis. The specificity difference between DBT and DxMM ranged from −10% to 23% across studies, while seven studies showed probably higher specificity for DBT. The visual assessment of the forest plots for each test independently showed a relevant heterogeneity for both DBT and DxMM (Figure S2). However, the specificity difference between tests showed a more homogenous pattern (Figure 3).

The pooled specificity was 74% (95% CI 69–78%) for DBT and 67% (95% CI 62–73%) for DxMM. The pooled difference was 6% (95% CI 2–11%; *p* = 0.001) in favor of DBT (Table 2). Considering the same 21% prevalence of BC lesions used for sensitivity, DBT probably reduces the false‐positive results by 47 (from 16 to 87 fewer) cases per 1000 women assessed (Table 3).

### 3.5 HSROC curve

**FIGURE 4:**
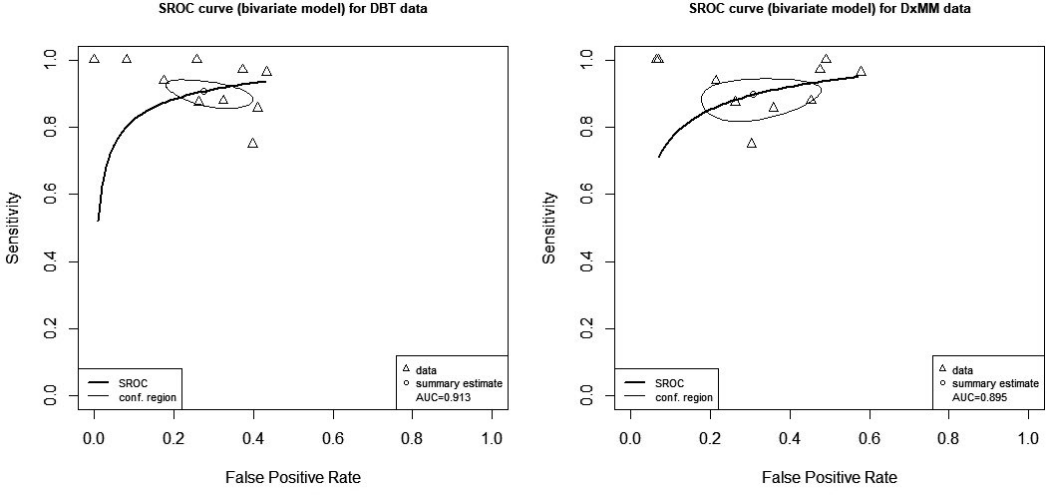
Hierarchical summary receiver operating characteristic (HSROC) curves for each test modality.

The larger the area under the curve (AUC) for the HSROC, the better the diagnostic performance. We found an AUC of 0.913 and 0.895 for DBT and DxMM, respectively (Figure 4), with overlapping confidence regions, which were wider for DxMM.

### 3.6 Subgroup and sensitivity analysis

Due to non‐convergence of the quadrivariate model with sparse data we only performed a sensitivity analysis for microcalcification lesions. Excluding the studies that did not include women with calcified lesions at screening mammography, we found a pooled difference of 5% (95% CI 1–9%) and 11% (95% CI 6–17%) for sensitivity and specificity, respectively (Table 2). Additionally, we did a sensitivity analysis excluding the study by Poplack et al., which had inconsistent results compared to the other included studies, but this did not change the original estimates.

Two studies reported accuracy estimates for patients with only calcified lesions. Michel et al, reported no significant differences among tests, with an area under the ROC curve of 79% (95% CI 73–86%) for DBT and of 78% (95% CI 72–85%) for DxMM.^9^ Gilbert et al., in a sample of 1027 cases with calcified lesions showed a sensitivity difference of −3% (95% CI −9% to 3%) and a specificity difference of 13% (95%CI 8–18%).^16^

### 3.7 Risk of bias and certainty of the evidence

In all the included studies there was a high or unclear risk of bias regarding the index test comparisons due to: (1) not clearly implementing a protocol to blind the assessment, the index tests or the assessors to the results of the histological or clinical exams,^9^, ^18^, ^23^ and (2) an imbalance in the experience of the reader of the index test results, as most of them had little training in the assessment of DBT images (ie, less than 1‐year experience or attending 1‐day training / 80 cases before the study)^16^, ^17^, ^18^, ^20^, ^21^, ^24^ or did not report the experience of DBT readers.^9^, ^19^, ^22^, ^23^

**FIGURE 5:**
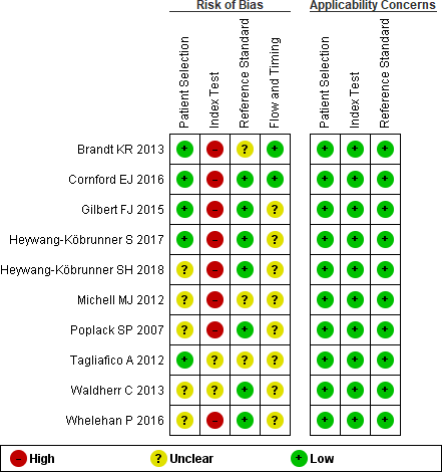
QUADAS‐2 risk of bias assessment.

Four studies inappropriately excluded participants with calcified lesions, something that could improve the accuracy of the index tests but of an unclear impact in the comparison. There were other limitations in the study designs, such as not having a prespecified threshold^9^, ^21^ or not including a clinical follow‐up for negative results, but these were similar in both index tests (Figure 5).

We considered that risk of bias due to these aspects was serious and therefore judged the certainty of the evidence as moderate. We observed some heterogeneity in the sensitivity and specificity differences but it was probably explained by the use of different thresholds to define positive results across studies and, to a lesser extent, due to the diversity of additional imaging tests or reference standards used (Table 3; Table S2).

## 4 DISCUSSION

### 4.1 Main findings

Our systematic review found that in women of average risk of BC with suspicious lesions at mammography screening, DBT probably has a higher specificity difference (6%) than diagnostic views of 2D mammography in women recalled for assessment (moderate certainty evidence). The sensitivity is also probably higher with DBT though in this case the difference was not so large (2%). The clinical impact of these results in a screening program is a probable reduction of 47 false‐positive results, including related invasive procedures, and most likely a small increase of 4 additional true BC lesions detected per 1000 women recalled for assessment. Our results were consistent in women with calcified lesions, but with a larger imprecision in the sensitivity differences. These results were taken into account by the GDG, together with other considerations (ie, feasibility) to issue a conditional recommendation in favor of DBT, available on the ECIBC website (https://healthcare‐quality.jrc.ec.europa.eu/european‐breast‐cancer‐guidelines/diagnosis/DBT)

### 4.2 Our results in the context of previous research

Previous studies assessing the role of DBT have focused on the screening setting. A prospective study from Europe reported a 40% improvement in the detection of invasive cancers and a 15% reduction in the number of false‐positive results.^25^ A systematic review in the screening setting for asymptomatic women, included 17 studies and found that tomosynthesis improves BC detection rate and reduces recall, with greater improvement in detection rate among European studies.^10^ Alabousi et al published a broader systematic review that identified 38 studies and reported that DBT alone or combined with DM had a higher sensitivity than DM alone (and also a higher specificity though smaller in magnitude). However, the authors included studies in symptomatic women and did not distinguish between the screening and diagnostic setting in the analysis.^26^

In contrast to the screening setting, the body of evidence on DBT in the assessment of screen‐recalled findings is scarcer. Li et al. published a systematic review in the diagnostic setting, which includes six studies suggesting that DBT may improve specificity but have no effect on sensitivity;^11^ as well as one of the included studies showing that DBT may reduce the frequency of ultrasound requests at assessment.^17^ Our findings are consistent with the observations from Li et al^11^ regarding specificity, however, we found a greater sensitivity for DBT. Additionally, we performed a more up‐to‐date search identifying five more studies and excluded one that did not compare DBT to additional views of DM but instead simulated the application of DBT as a triage tool.^27^

It is worth noting that our findings are consistent with a recent study suggesting the use of DBT as an add‐on test, although this study used DBT in a different diagnostic pathway.^28^ Sharma et al. compared triple assessment (further 2D mammography views, clinical examination, and ultrasound) versus triple assessment plus DBT among women recalled at screening within the UK National Health Service Breast Screening Programme.^28^ The addition of DBT resulted in similar sensitivity but higher specificity (38.2% vs 77.5%), which translated into a reduction in the number of biopsies from 571 of 827 (69.0%) to 298 of 827 (36.0%).^28^

In most of the included studies in our review, the readers interpreted the DBT images having the screening mammography available to them. One study used synthetic mammography (SM) in combination with DBT showing results consistent with other studies. This may suggest a potential use of SM in the assessment of abnormalities at recall.^16^ In a different population, Mumin et al, compared the accuracy of SM+DBT to that of DM+DBT, in women referred for assessment due to the presence of symptoms, finding a high agreement between the use of DM or SM in combination with DBT.^29^

### 4.3 Limitations and strengths

Our systematic review has some limitations. We included only articles in English, but the risk of selection bias is probably small because we screened previous systematic reviews, and the GDG includes several international experts in the field. Most studies had limitations: (1) In many studies the radiologist readers had lower experience in DBT compared to DM, (2) the work‐up in the included studies was not limited to mammography or DBT but also used other imaging modalities (ie, breast ultrasound), which might have hidden true differences between the index tests, and (3) some studies did not implement explicit blinding of readers during the assessment and used imperfect reference standards that might include or not clinical follow‐up.

Additionally, the included studies did not use the same thresholds to interpret the results of the index tests or did not clearly define the threshold, referring only to the standard clinical practice. However, we considered that it was appropriate to provide pooled estimates because (1) the reported definitions were to some degree clinically equivalent (ie, UK‐MRS 3 to 5 vs BI‐RADS 3 to 5) and (2) our interest was not to determine the accuracy of each testing strategy but to assess the difference between them, and all studies included a paired design.

Our review has several strengths. The review was developed as part of a clinical guideline development process and the GDG also evaluated DBT as a primary screening test guaranteeing a broader view of the evidence. The clinical question was framed considering the complete screening and diagnostic pathway, thus placing it in context with the whole work‐up process and the clinical utility of the results. We included studies with a paired design to directly compare both index tests. We identified studies that recruited participants from routine screening programs which makes our results robust in terms of applicability to the clinical practice. We also used recommended statistical methods to pool accuracy studies and included the GRADE approach to rate the certainty of the evidence, considering the comparative framework of our question of interest.

### 4.4 Implications for practice and research

Our findings may have different implications for practice depending on the stakeholder group. In the case of women, it would depend on how they value the balance between potential benefits and harms derived from the replacement of diagnostic mammography projections with DBT in the assessment setting. Thus, in a society where women are more concerned about false‐positive results or invasive procedures derived from false‐positive results, in the context of a shared decision‐making process, women might be more in favor of DBT. In the case of guideline panels and policymakers, they might consider other aspects such as the use of resources or feasibility issues around implementing this technology which could influence their decision to formulate a strong or conditional recommendation.

The available body of evidence for the use of DBT was of moderate certainty due to risk of bias, thus there is a need to produce further evidence from implementation data of this technology in the assessment context. Among the research priorities identified during this review, with input from the GDG experts, are the following: i) given that ultrasound is often included in the management of assessment after a positive finding in screening mammography, further research should be conducted exploring which subgroups could avoid ultrasound after DBT‐additional projections, as well as which lesions (ie, masses) could be assessed with ultrasound instead of additional mammography projections or DBT; ii) Use of DBT in women with high mammographic breast density, particularly for the assessment of abnormalities at screening examination, and iii) whether or not to use one or two views for tomosynthesis in the assessment.

## CONFLICT OF INTEREST

Zuleika Saz‐Parkinson and Elena Parmelli were or are current employees of the Joint Research Centre, European Commission. Carlos Canelo‐Aybar, Jessica Beltrán, David Rigau, Margarita Posso, and Pablo Alonso‐Coello, are employees of the Iberoamerican Cochrane Collaboration. Dr. Giorgi Rossi as former‐PI of an independent study on HPV‐based cervical cancer screening, funded by the Italian Ministry of Health, data owner, conducted negotiations with Roche diagnostics, Hologic‐Genprobe, Becton‐Dickinson to obtain reagents at reduced price or for free; the reagents obtained were not used in his institution. Dr. Lebeau reports grants and reimbursement for travel‐related expenses related to consultancy from Roche Pharma AG, reimbursement for travel‐related expenses related to consultancy from Novartis Oncology, and grants from BioNTech Diagnostics GmbH outside the submitted work. Dr. Gräwingholt is the responsible radiologist for screening unit Paderborn, Germany, consultant radiologist for screening programs in Switzerland, and consultant radiologist for Hellenic School of Senology. Annette Lebeau, Axel Gräwingholt, Xavier Castells, Miranda Langendam, Elsa Pérez, Paolo Giorgi Rossi, and Ruben Van Engen are members of the ECIBC Guidelines Development Group.

## AUTHORS' CONTRIBUTIONS

Carlos Canelo‐Aybar, Lourdes Acosta, and Pablo Alonso‐Coello were responsible for conducting the systematic review conducted the search and data extraction. Paolo Giorgi Rossi, Zuleika Saz‐Parkinson, and Axel Gräwingholt contributed to the definition of the research protocol and provided comments to the preliminary results of the systematic review. Carlos Canelo‐Aybar drafted the first version of the article. All authors contributed to the interpretation and reporting of the results and provided comments on subsequent versions of the article. All authors read and approved the final manuscript prior submission.

## Supporting information

Supplementary Material

## Data Availability

All data sources used during this study are described in this published article and its additional information files. The datasets analyzed are available from the corresponding author on reasonable request.
