# Supplementary material for: Digital breast tomosynthesis compared to diagnostic mammographic projections (including magnification) among women recalled at screening mammography: a systematic review for the European Commission Initiative on Breast Cancer (ECIBC)
Source: Cancer Med. 2021 Mar 5;10(7):2191–204. doi: 10.1002/cam4.3803 (PMC7982617; doi:10.1002/cam4.3803)
Supplement: Supplementary file 1 — Supplementary Material [file CAM4-10-2191-s001.docx]

**Supplementary file**

**Table S1.** **Search strategy for the evidence of effects**

| **Clinical question**  Should digital breast tomosynthesis (DBT) vs diagnostic mammography projections (including magnification) be used in the assessment of recalled women of average risk of breast cancer due to suspicious lesions at mammography screening? | |
| --- | --- |
| **MEDLINE**  PubMed  15/02/2018 | #1        "Breast Neoplasms"[Mesh]     (257687)  #2        breast*[ti]      (245003)  #3        #1 OR #2       (322294)  #4        “Radiographic Image Enhancement”[Mesh]       (376921)  #5        tomosynthes*[tiab]       (1194)  #6        DBT[tiab]        (1962)  #7        3D-mammograph*[tiab] (29)  #8        #4 OR #5 OR #6 OR #7  (379090)  #9        #3 AND #8        (5683)  #10       “diagnostic imaging”[sh] (1010048)  #11       diagnos*[tiab]    (2140322)  #12       recall*[tiab]        (59285)  #13       abnormal*[tiab]  (638238)  #14       #10 OR #11 OR #12 OR #13       (3399507)  #15       #9 AND #14      (4432)  #16       "Early Detection of Cancer"[Mesh]        (16946)  #17      #15 NOT #16    (4262) |
| **The Cochrane Library**  04/05/2018 | #1 MeSH descriptor: [Breast Neoplasms] explode all trees (10718)  #2 breast*:ti,ab,kw (Word variations have been searched) (34413)  #3 #1 or #2 (34413)  #4 MeSH descriptor: [Radiographic Image Enhancement] explode all trees (5812)  #5 tomosynthes*:ti,ab,kw (Word variations have been searched) (54)  #6 DBT:ti,ab,kw (Word variations have been searched) (205)  #7 3D-mammograph*:ti,ab,kw (Word variations have been searched) (2)  #8 #4 or #5 or #6 or #6 or #7 (6037)  #9 #3 and #8 (162)  #10 MeSH descriptor: [Diagnostic Imaging] explode all trees (48800)  #11 diagnos*:ti,ab,kw (Word variations have been searched) (102563)  #12 recall*:ti,ab,kw (Word variations have been searched) (8168)  #13 abnormal*:ti,ab,kw (Word variations have been searched) (23775)  #14 #10 or #11 or #12 or #13 (166967)  #15 #9 and #14 (160)  #16 MeSH descriptor: [Early Detection of Cancer] explode all trees (1126)  #17 #15 not #16 (153) |
| **EMBASE**  Ovid Embase <1980 to 2016 Week 16>  15/02/2018 | 1 exp breast tumor/ (448712)  2 "breast*".ab,ti. (511507)  3 1 or 2 (607574)  4 exp digital breast tomosynthesis/ (480)  5 "tomosynthes*".ab,ti. (1471)  6 DBT.ab,ti. (2647)  7 "3D-mammograph*".ab,ti. (39)  8 4 or 5 or 6 or 7 (3764)  9 3 and 8 (1064)  10 "diagnos*".ab,ti. (2858462)  11 "recall*".ab,ti. (74244)  12 "abnormal*".ab,ti. (802602)  13 suspicious.ab,ti. (32168)  14 exp diagnostic imaging/ (150021)  15 exp diagnostic accuracy/ (exp diagnostic accuracy/ (220347)  16 exp "sensitivity and specificity"/ (284981)  17 10 or 11 or 12 or 13 or 14 or 15 or 16 or 17 (3817486)  18 9 and 17 (663) |

**Figure S1.** Forest plots for sensitivity of digital breast tomosynthesis and diagnostic mammography


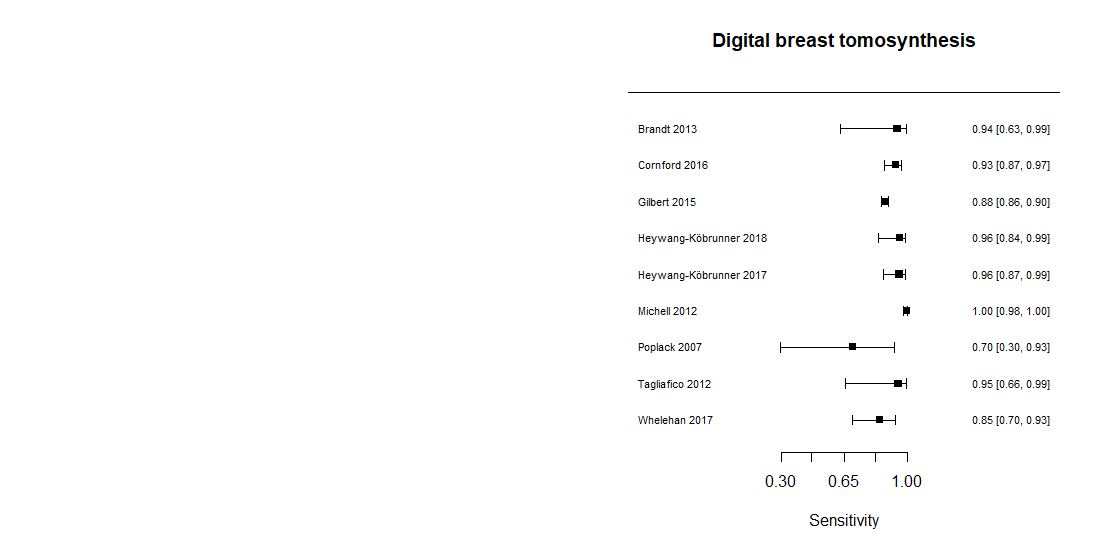

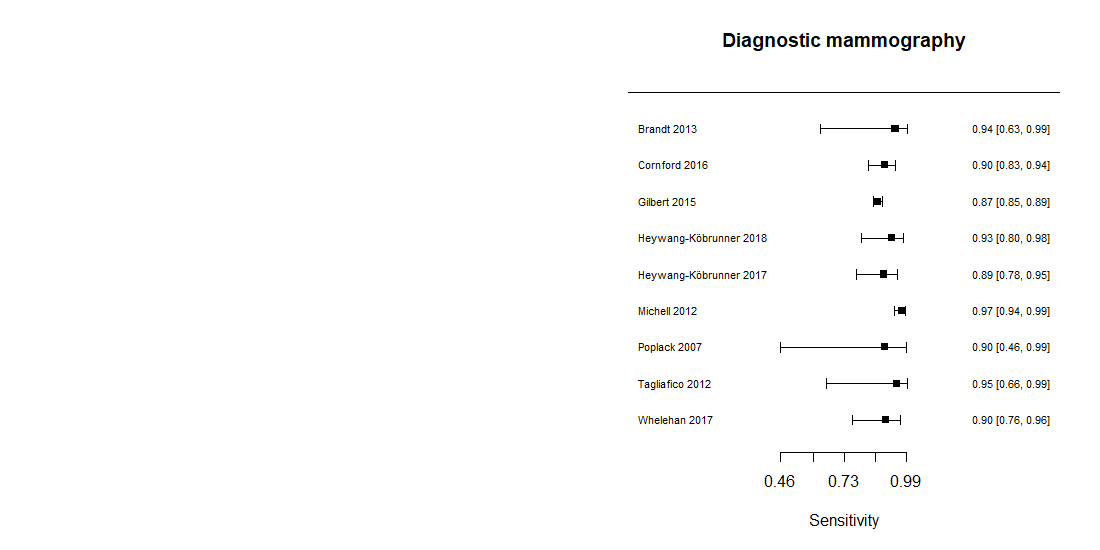


**Figure S2**. Forest plots for specificity of digital breast tomosynthesis and diagnostic mammography


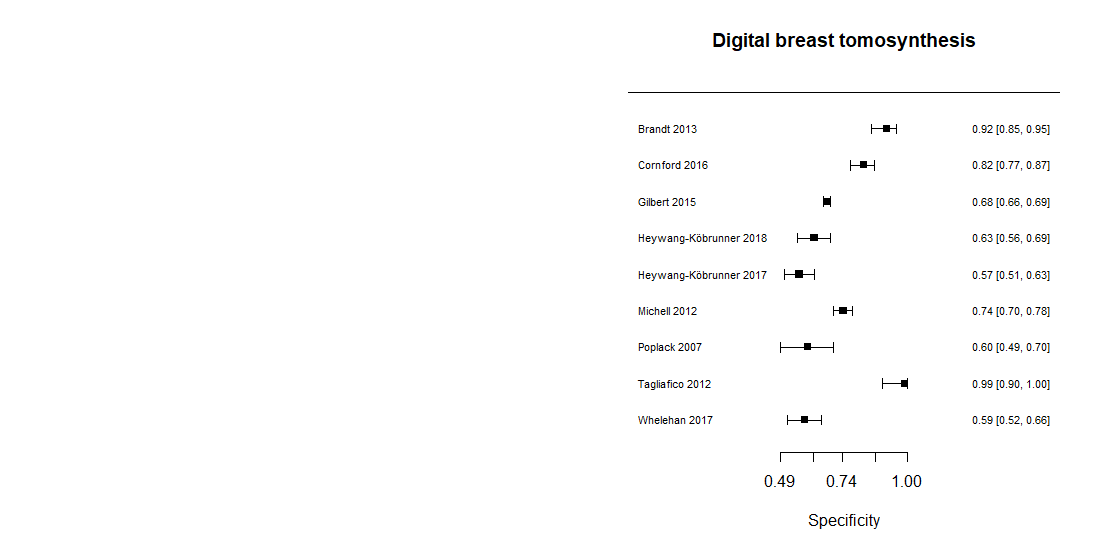

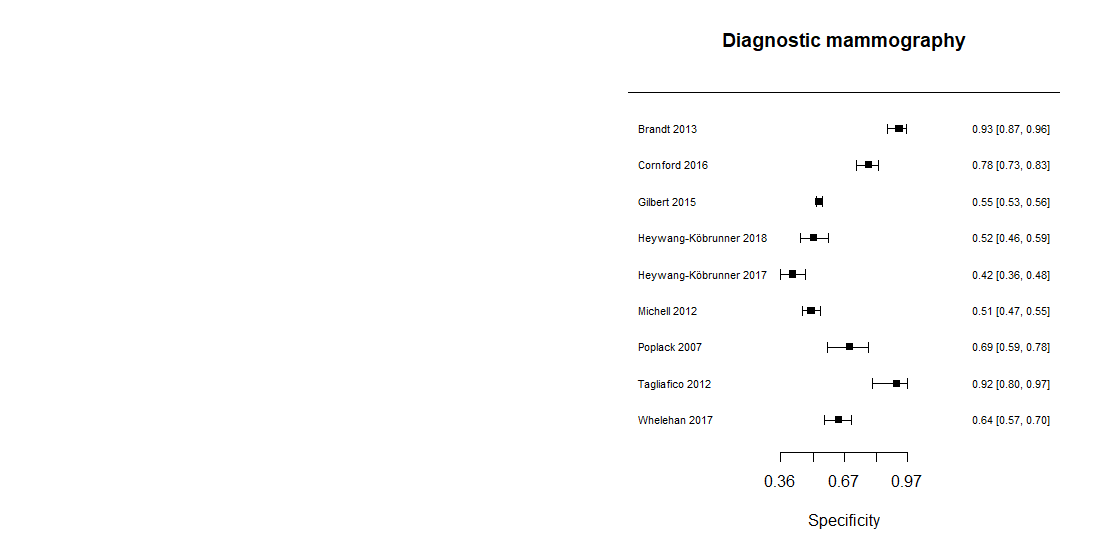


**Table S2. Evidence profile**

| \| Digital breast tomosynthesis \| \| Diagnostic mammography \| \| Pooled difference \| \| \| --- \| --- \| --- \| --- \| --- \| --- \| \| Sensitivity \| 0.94 (95% CI: 0.91 to 0.97) \| Sensitivity \| 0.92 (95% CI: 0.89 to 0.95) \| Sensitivity \| 0.02 (95% CI:0.01 to 0.03) \| \| Specificity \| 0.74 (95% CI: 0.69 to 0.78) \| Specificity \| 0.67 (95% CI: 0.62 to 0.73) \| Specificity \| 0.06 (95% CI: 0.02 to 0.11) \| |  | \| Prevalences \| 21% \| 9% \| \| --- \| --- \| --- \| |  |
| --- | --- | --- | --- | --- | --- | --- | --- | --- | --- | --- | --- | --- | --- | --- | --- | --- | --- | --- | --- | --- | --- | --- | --- | --- |

| Outcome | № of studies (№ of patients) | Study design | Factors that may decrease certainty of evidence | | | | | Effect per 1000 patients tested | | | | Test accuracy CoE |
| --- | --- | --- | --- | --- | --- | --- | --- | --- | --- | --- | --- | --- |
|  |  |  |  |  |  |  |  | pre-test probability of 21% | | pre-test probability of 9% | |  |
|  |  |  | Risk of bias | Indirectness | Inconsistency | Imprecision | Publication bias | digital breast tomosynthesis (paper) | assessment mammography | digital breast tomosynthesis (paper) | assessment mammography |  |
| **True positives** | 10 studies 1584 patients ^1,2,3,4,5,6,7,8,9,10,a^ | cross-sectional (cohort type accuracy study) | serious ^b^ | not serious ^c^ | not serious ^d,e^ | not serious | none ^f^ | 197 (191 to 204) | 193 (187 to 199) | 85 (82 to 87) | 83 (80 to 85) | ⨁⨁⨁◯ MODERATE |
|  |  |  |  |  |  |  |  | **4 more TP in digital breast tomosynthesis** | | **2 more TP in digital breast tomosynthesis** | |  |
| **False negatives** |  |  |  |  |  |  |  | 13 (6 to 19) | 17 (11 to 23) | 5 (3 to 8) | 7 (5 to 10) |  |
|  |  |  |  |  |  |  |  | **4 fewer FN in digital breast tomosynthesis** | | **2 fewer FN in digital breast tomosynthesis** | |  |
| **True negatives** | 10 studies 6096 patients ^1,2,3,4,5,6,7,8,9,10,a^ | cross-sectional (cohort type accuracy study) | serious ^b^ | not serious ^c^ | not serious ^g,h^ | not serious | none ^f^ | 585 (545 to 616) | 529 (490 to 577) | 673 (628 to 710) | 610 (564 to 664) | ⨁⨁⨁◯ MODERATE |
|  |  |  |  |  |  |  |  | **47 more TN in digital breast tomosynthesis** | | **55 more TN in digital breast tomosynthesis (paper)** | |  |
| **False positives** |  |  |  |  |  |  |  | 205 (174 to 245) | 261 (213 to 300) | 237 (200 to 282) | 300 (246 to 346) |  |
|  |  |  |  |  |  |  |  | **47 fewer FP in digital breast tomosynthesis** | | **55 fewer FP in digital breast tomosynthesis** | |  |

#### Explanations

a. The absolute differences are the additional cases identified or missed with digital breast tomosynthesis compared to additional mammographic views among those women recalled at the screening mammography assessment.

b. In some of the included studies, there was a non-blinded reading of the index tests. There was variability in how the evaluations were performed, in some cases, they included additional tests such as ultrasound. Those additional exams might be requested at clinical discretion and therefore could be a source of differential misclassification in the test accuracy estimates.

c. One study (Gilbert 2015), performed a retrospective analysis with DBT plus synthesized two-view mammography as the intervention index test. In the remaining studies, the intervention index test was DBT plus screening mammography.

d. There was no important unexplained heterogeneity in the sensitivity´s difference between test.

e. One study (Poplack 2012) showed largely inconsistent results. A sensitivity analysis excluding this study did not show relevant differences in the pooled estimate.

f. There was no evidence suggesting publication bias as far as the expert´s panel knowledge.

g. There was heterogeneity in the specificity´s difference between the index tests across studies.

h. Observed heterogeneity might be explained by a combination of studies` characteristics as the use of different thresholds to define positivity, use of diverse additional imaging tests or reference standards (none fully driving the heterogeneity across studies). The panel decided not to downgrade the certainty of evidence.

#### References

1. Tagliafico, A., Astengo, D., Cavagnetto, F., Rosasco, R., Rescinito, G., Monetti, F., Calabrese, M.. One-to-one comparison between digital spot compression view and digital breast tomosynthesis. Eur Radiol; Mar 2012.

2. Heywang-Köbrunner S, Jaensch A,Hacker A,Wulz-Horber S,Mertelmeier T,Hölzel D.. Value of Digital Breast Tomosynthesis versus Additional Views for the Assessment of Screen-Detected Abnormalities - a First Analysis. Breast Care (Basel); 2017.

3. Whelehan, P., Heywang-Kobrunner, S. H., Vinnicombe, S. J., Hacker, A., Jansch, A., Hapca, A., Gray, R., Jenkin, M., Lowry, K., Oeppen, R., Reilly, M., Stahnke, M., Evans, A.. Clinical performance of Siemens digital breast tomosynthesis versus standard supplementary mammography for the assessment of screen-detected soft-tissue abnormalities: a multi-reader study. Clinical radiology; 2017.

4. Poplack, S. P., Tosteson, T. D., Kogel, C. A., Nagy, H. M.. Digital breast tomosynthesis: initial experience in 98 women with abnormal digital screening mammography. AJR Am J Roentgenol; Sep 2007.

5. Heywang-Kobrunner, S. H., Hacker, A., Jansch, A., Kates, R., Wulz-Horber, S., German Reader, Team. Use of single-view digital breast tomosynthesis (DBT) and ultrasound vs. additional views and ultrasound for the assessment of screen-detected abnormalities: German multi-reader study. Acta Radiol; Jan 1 2017.

6. Gilbert, F. J., Tucker, L., Gillan, M. G., Willsher, P., Cooke, J., Duncan, K. A., Michell, M. J., Dobson, H. M., Lim, Y. Y., Suaris, T., Astley, S. M., Morrish, O., Young, K. C., Duffy, S. W.. Accuracy of Digital Breast Tomosynthesis for Depicting Breast Cancer Subgroups in a UK Retrospective Reading Study (TOMMY Trial). Radiology; Dec 2015.

7. Cornford, E. J., Turnbull, A. E., James, J. J., Tsang, R., Akram, T., Burrell, H. C., Hamilton, L. J., Tennant, S. L., Bagnall, M. J., Puri, S., Ball, G. R., Chen, Y., Jones, V.. Accuracy of GE digital breast tomosynthesis vs supplementary mammographic views for diagnosis of screen-detected soft-tissue breast lesions. Br J Radiol; 2016.

8. Brandt, K. R., Craig, D. A., Hoskins, T. L., Henrichsen, T. L., Bendel, E. C., Brandt, S. R., Mandrekar, J.. Can digital breast tomosynthesis replace conventional diagnostic mammography views for screening recalls without calcifications? A comparison study in a simulated clinical setting. AJR Am J Roentgenol; Feb 2013.

9. Michell MJ, Iqbal A,Wasan RK,Evans DR,Peacock C,Lawinski CP,Douiri A,Wilson R,Whelehan P.. A comparison of the accuracy of film-screen mammography, full-field digital mammography, and digital breast tomosynthesis. Clin Radiol; 2012.

10. Waldherr, C., Cerny, P., Altermatt, H. J., Berclaz, G., Ciriolo, M., Buser, K., Sonnenschein, M. J.. Value of one-view breast tomosynthesis versus two-view mammography in diagnostic workup of women with clinical signs and symptoms and in women recalled from screening. AJR Am J Roentgenol; Jan 2013.

**Table S3. List of excluded studies and reasons for exclusions**

| **Individual excluded studies (original search)** | | |
| --- | --- | --- |
| **Author** | **Year** | **Reason for exclusion** |
| 1. Bansal | 2015 | Different population: symptomatic women |
| 1. Bernanrdi | 2012 | No comparison to diagnostic mammography |
| 1. Carbonaro | 2016 | No comparison to diagnostic mammography |
| 1. Cavagnetto | 2013 | Screening setting |
| 1. Chae | 2016 | Different population: symptomatic and asymptomatic women |
| 1. Chou | 2015 | Different tests: contrast enhanced exams |
| 1. Ekpo | 2014 | Different design: systematic review |
| 1. Elizalde | 2016 | Different comparison: ultrasound |
| 1. Endo | 2017 | Different population: screening and diagnostic population |
| 1. Endo | 2016 | Different intervention: FFDM plus DBT, no 2x2 results. |
| 1. Forvinkm | 2010 | Different population: symptomatic and asymptomatic women |
| 1. Genaro | 2010 | Different population: recalled by mammography or ultraosound |
| 1. Gur | 2012 | Different population: no recalled from screening |
| 1. Hakim | 2010 | Different population: recalled from screening or women required to perform a biopsy |
| 1. Hunter | 2017 | Screening setting |
| 1. Kang | 2016 | Different population: screening and diagnostic population |
| 1. Haq | 2015 | Compared with screening mammography |
| 1. Kim | 2017 | Different population: screening and diagnostic population (dense breast) |
| 1. Krammer | 2017 | Different population: invasive breast cancer under staging assessment |
| 1. Lei | 2013 | Different design: systematic review |
| 1. Mansour | 2014 | Different population: symptomatic and asymptomatic women |
| 1. Mariscotti | 2016 | Different population: invasive breast cancer under staging assessment |
| 1. Maxwell | 2017 | Screening setting |
| 1. Mcdonald | 2017 | Screening setting |
| 1. Morel | 2014 | Different population: symptomatic and asymptomatic women |
| 1. Sumkin | 2015 | Screening setting |
| 1. Svahn | 2012 | Different population: symptomatic and asymptomatic women |
| 1. Savane | 2011 | Different population: screening, symptomatic and asymptomatic women |
| 1. Takamoto | 2014 | Different population: invasive breast cancer under staging assessment |
| 1. Teertstra | 2010 | Different population: symptomatic and asymptomatic women |
| 1. Thibault | 2013 | Different population: symptomatic and asymptomatic women |
| 1. Tucker | 2017 | Data analysis duplicate of tommy trial |
| **Individual excluded studies (update search on February, 2020)** | | |
| 1. Alkhalik |  | Different population: symptomatic and asymptomatic women |
| 1. Alshafeiy | 2018 | Different population: screening and diagnostic population |
| 1. Choi | 2018 | Different design: case control |
| 1. Dodelzon |  | Different design: case control |
| 1. Garayoa | 2017 | No comparison to diagnostic mammography |
| 1. Georgian-Smith | 2019 | Different design: multi case study |
| 1. Giess | 2017 | Different population: screening assessment |
| 1. Hawley | 2017 | Different population: symptomatic |
| 1. Honing | 2019 | Different setting: screening mammography |
| 1. Lee | 2019 | Different setting: no recall setting |
| 1. Munin | 2019 | Different setting: no recall setting |
| 1. Sharma | 2019 | Different intervention: DBT plus diagnostic mammography together |
| 1. Singla | 2019 | Different setting: no recall setting |
| 1. Stepanek | 2019 | Different setting: screening mammography |
| 1. Viajpura | 2018 | Different setting: screening mammography |
| 1. Yi | 2018 | Different setting: no recall setting |
